# Right ventriculo–arterial uncoupling and impaired contractile reserve in obese patients with unexplained exercise intolerance

**DOI:** 10.1007/s00421-018-3873-4

**Published:** 2018-04-30

**Authors:** Colm McCabe, Rudolf K. F. Oliveira, Farbod Rahaghi, Mariana Faria-Urbina, Luke Howard, Richard G. Axell, Andrew N. Priest, Aaron B. Waxman, David M. Systrom

**Affiliations:** 1 Division of Cardiology, Royal Brompton Hospital, London, SW3 6NP UK; 2 Division of Pulmonary and Critical Care Medicine, Department of Medicine, Brigham and Women’s Hospital and Harvard Medical School, Boston, USA; 3 Hammersmith Hospital, London, UK; 4 Addenbrookes Hospital, Cambridge, UK; 5 Division of Respiratory Diseases, Department of Medicine, Federal University of São Paulo (UNIFESP), São Paulo, SP Brazil

**Keywords:** Obesity, Exercise, Right ventricle, Coupling, Afterload

## Abstract

**Background:**

Right ventricular (RV) dysfunction and heart failure with preserved ejection fraction may contribute to exercise intolerance in obesity. To further define RV exercise responses, we investigated RV–arterial coupling in obesity with and without development of exercise pulmonary venous hypertension (ePVH).

**Methods:**

RV–arterial coupling defined as RV end-systolic elastance/pulmonary artery elastance (Ees/Ea) was calculated from invasive cardiopulmonary exercise test data in 6 controls, 8 obese patients without ePVH (Obese−ePVH) and 8 obese patients with ePVH (Obese+ePVH) within a larger series. ePVH was defined as a resting pulmonary arterial wedge pressure < 15 mmHg but ≥ 20 mmHg on exercise. Exercise haemodynamics were further evaluated in 18 controls, 20 Obese−ePVH and 17 Obese+ePVH patients.

**Results:**

Both Obese−ePVH and Obese+ePVH groups developed exercise RV–arterial uncoupling (peak Ees/Ea = 1.45 ± 0.26 vs 0.67 ± 0.18 vs 0.56 ± 0.11, *p* < 0.001, controls vs Obese−ePVH vs Obese+ePVH respectively) with higher peak afterload (peak Ea = 0.31 ± 0.07 vs 0.75 ± 0.32 vs 0.88 ± 0.62 mL/mmHg, *p* = 0.043) and similar peak contractility (peak Ees = 0.50 ± 0.16 vs 0.45 ± 0.22 vs 0.48 ± 0.17 mL/mmHg, *p* = 0.89). RV contractile reserve was highest in controls (ΔEes = 224 ± 80 vs 154 ± 39 vs 141 ± 34% of baseline respectively, *p* < 0.001). Peak Ees/Ea correlated with peak pulmonary vascular compliance (PVC, *r* = 0.53, *p* = 0.02) but not peak pulmonary vascular resistance (PVR, *r* = − 0.20, *p* = 0.46). In the larger cohort, Obese+ePVH patients on exercise demonstrated higher right atrial pressure, lower cardiac output and steeper pressure-flow responses. BMI correlated with peak PVC (*r* = − 0.35, *p* = 0.04) but not with peak PVR (*r* = 0.24, *p* = 0.25).

**Conclusions:**

Exercise RV–arterial uncoupling and reduced RV contractile reserve further characterise obesity-related exercise intolerance. RV dysfunction in obesity may develop independent of exercise LV filling pressures.

## Introduction

Heart failure with preserved ejection fraction (HFpEF) and right ventricular (RV) dysfunction are common in symptomatic obesity and can make a significant contribution toward exercise symptoms. Both predispose obese patients to further impairment in left ventricular (LV) filling on exercise, greater risk of biventricular remodelling and more severe haemodynamic derangement (Alpert et al. 2014). Independent of HFpEF and sleep disordered breathing, obesity has also been associated with increased RV mass, higher RV end-diastolic volumes and reduced RV systolic function (Chahal et al. 2012; Wong et al. 2006). This suggests that obesity itself may carry specific predisposition to RV dysfunction and that, in cases where resting haemodynamics do not explain the level of exercise intolerance, pulmonary haemodynamic evaluation during exercise may be used to unmask obesity-related pulmonary vascular and RV dysfunction (Chahal et al. 2012).

We hypothesised that obesity itself invokes a direct negative influence on the RV exercise contractile response through greater thoracic mechanical loading and higher exercise RV afterload. To measure RV contractile and afterload responses, we evaluated RV end-systolic elastance (Ees) and pulmonary arterial elastance (Ea) respectively, to derive RV–arterial coupling (Ees/Ea) ratios. RV and pulmonary arterial elastance were derived from resting and exercise RV pressure–volume relationships drawn directly from invasive cardiopulmonary exercise test data of obese patients undergoing investigation of unexplained exercise intolerance. To account for the influence of impaired LV filling on exercise in obesity which may increase RV exercise afterload, RV elastance, pulmonary arterial elastance and Ees/Ea ratios were measured in two groups of symptomatic obese patients demonstrating either normal or elevated LV filling pressures on exercise. Their exercise data were compared to a third group of non-obese controls also undergoing evaluation of unexplained dyspnoea. In a larger cohort of 37 obese patients and 18 controls drawn from the same referral pool, we examined relationships between BMI and exercise haemodynamics.

## Methods

### Study population

All patients included in the study were derived from a cohort of over 750 sequential patients referred to Brigham and Women’s Hospital for unexplained exertional intolerance. All completed invasive cardiopulmonary exercise testing for clinical investigation of dyspnoea, after resting lung function, echocardiography and routine imaging had not uncovered a cause for the patient’s symptoms. Imaging undertaken prior to invasive cardiopulmonary exercise testing included CT pulmonary angiography and/or ventilation perfusion scintigraphy to exclude thromboembolic disease as well as high resolution thoracic CT to evaluate for the presence of interstitial lung disease. Following review of available invasive exercise test data, three patients groups were identified: one obese group with exercise pulmonary venous hypertension (ePVH, Obese+ePVH), one obese group without ePVH (Obese−ePVH) and a group of non-obese patients with exertional intolerance, but normal exercise physiology (controls). Obese patients were defined by a body mass index (BMI) > 30 kg/m^2^.

#### Exclusion criteria and group derivations

Patients were excluded from study analysis by several factors. These consisted of failure to demonstrate maximal effort on exercise testing defined by a respiratory exchange ratio (RER) > 1.05 and peak HR > 90% predicted, the presence of moderate or worse valvular heart disease or reduced resting left ventricular ejection fraction (< 50%) judged from a contemporary resting transthoracic echocardiogram undertaken within 3 months of the exercise test, presence of clinically significant connective tissue disease, interstitial lung disease or thromboembolic disease, prior cardiac or lung transplantation, smoking pack year history of > 20 or a resting FEV_1_/FVC ratio < 70% on spirometry. Control patients were selected based on a normal exercise capacity defined by a peak *V*O_2_ > 80% predicted (Hansen et al. 1984), a normal cardiac output response to exercise (> 80% predicted) and a Ca-vO_2_ content difference /Hb > 80%.

Obese patients with ePVH (Obese+ePVH, *n* = 8) were defined by having a resting pulmonary arterial wedge pressure (PAWP) ≤ 15 and ≥ 20 mmHg at peak exercise based on historical cut-offs for exercise LV filling pressure (Groves et al. 1987; Parker and Thadani 1979; Wagner et al. 1986). Obese patients without ePVH (Obese−ePVH, *n* = 8) were defined by a resting PAWP ≤ 15 mmHg and an exercise PAWP < 20 mmHg. No control patients demonstrated an elevated PAWP either at rest or on exercise.

**Fig. 1:**
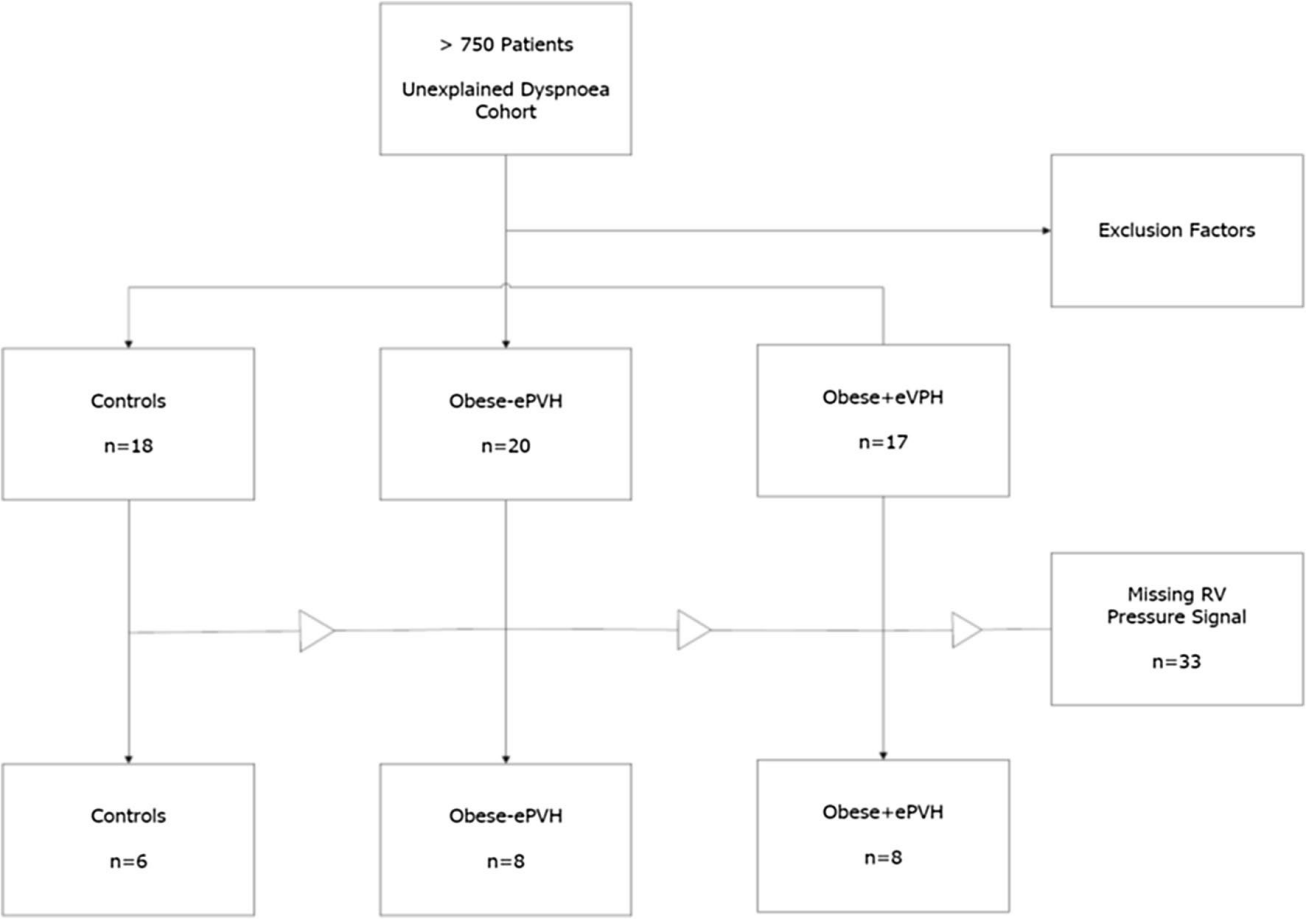
Flowchart showing identification of control, Obese−ePVH and Obese+ePVH groups derived from a larger population investigated for unexplained dyspnoea

The derivation of control, Obese−ePVH and Obese+ePVH groups is further outlined in Fig. 1. Out of over 750 sequential patients, 37 obese patients met criteria for study inclusion. From these 37 patients, 16 had complete RV pressure waveform data between rest and peak exercise enabling calculation of RV–arterial coupling parameters (Ees, Ea, Ees/Ea). 16 controls met criteria for study inclusion and of these, 6 patients had similarly complete RV pressure waveform data between rest and peak exercise. In the remaining controls and obese patients not evaluated for RV–arterial coupling, the transduced signal from the proximal RV catheter port was either entirely missing due to patient anatomical factors or was temporarily lost during the exercise test rendering their contractility and afterload analysis incomplete. These patients’ data were incorporated into the analysis of BMI and exercise haemodynamics.

#### Invasive cardiopulmonary exercise testing

The full protocol for invasive cardiopulmonary exercise testing and its analysis was undertaken as previously described (Maron et al. 2013). Briefly, a pulmonary artery catheter was placed in the Brigham and Women’s Hospital cardiac catheterization lab as per standard procedure via the internal jugular vein with ultrasound and fluoroscopic guidance. The pulmonary artery catheter was a flow-directed, balloon-tipped, 4-port pacing pulmonary arterial catheter (Edwards Lifesciences, Irvine, CA, USA). An arterial line was inserted into the radial artery using a 20-gauge angiocatheter or 5-French sheath. All exercise tests were performed in the Brigham and Women’s Hospital cardiopulmonary exercise laboratory located adjacent to the cardiac catheterization laboratory. All subjects completed a single bout of incremental cycling to exhaustion on an upright ergometer (Medgraphics Corival Cycle Ergometer, Medical Graphics Corp, St. Paul, MN, USA). The tests were performed with the patient breathing room air. At least 2 min of rest was followed by 3 min of unloaded cycling at 60–70 revolutions per minute. Work was then continuously increased using a ramp protocol by 5, 10, 15, or 20 W/min based on historic exercise tolerance in the field to obtain a test duration between 8 and 10 min. After reaching a symptom-limited maximum, patients entered a recovery period.

Pulmonary gas exchange was measured breath-by-breath. Heart rate (HR), systemic systolic blood pressure (SBP), systemic diastolic blood pressure (DBP), right atrial (RAP), right ventricular, and pulmonary artery (PAP) pressures were measured continuously using a Phillips Xper Cardio Physiomonitoring System (Andover, MA, USA) that was calibrated, levelled, and zeroed at the level of the right atrium before each study. At rest and during each minute of exercise, PAWP was measured as a calculated electronic mean over the respiratory cycle (Boerrigter et al. 2014), and a 12-lead electrocardiogram was obtained. One-millilitre blood samples were simultaneously drawn from the radial arterial catheter and distal port of the non-wedged pulmonary arterial catheter once every minute during rest and during the last 15 s of each minute during exercise. Axillary temperature-adjusted systemic arterial and mixed venous blood samples were analysed for partial pressure of oxygen (PaO_2_ or PvO_2_), partial pressure of carbon dioxide (PaCO_2_ or PvCO_2_), pH (Model 1620, Instrumentation Laboratories, Lexington, MA), bicarbonate concentration (HCO^3−^), haemoglobin concentration (Hb), oxygen saturation (SaO_2_ or SvO_2_) (Model 482, Instrumentation Laboratories), oxygen content (CaO_2_ or CvO_2_) by co-oximetry and serum lactate concentration.

#### Data analysis

Ventilatory and pulmonary gas exchange data were averaged over the final 30-s interval of the 2-min rest period and averaged over contiguous 30-s intervals during exercise. *V*O_2_ peak was defined as the highest 30-s averaged *V*O_2_ during the last minute of the symptom-limited exercise test. Predicted values of *V*O_2_ peak were controlled for age, gender, and height. Cardiac output (CO) was calculated from the direct Fick method (CO = *V*O_2_/[Ca-vO_2_]) and predicted CO at peak exercise was calculated from predicted *V*O_2_peak using an assumed maximal Ca-vO_2_ equivalent to a normal Hb (14 g/dL) for healthy subjects. Maximum voluntary ventilation was taken as resting FEV_1_ × 35. All invasive exercise test data were retrospectively reviewed.

### RV waveform analysis

To obtain RV–arterial coupling data, RV pressure tracings were obtained in the upright position at rest and at maximum effort and analysed using the PHILIPS Xper Information Management Software (Version 1.5.7.1850, Koninklijke Philips Electronics N.V. Amsterdam, The Netherlands). Pressure waveform tracings were recorded for 7 s duration and for at least 5 cardiac cycles. Numerical data were extracted by conversion of binary data into 16-bit integers using MATLAB R2016a (Mathworks, Natick, MA, USA). Each waveform was compared with RV pressure tracings extracted from real-time haemodynamic monitoring to verify true integrity of the data. The timings of 7 s captures were matched to mean stroke volumes obtained during 7 s capture to calculate pressure–volume relationships.

To calculate Ees, the single-beat method was employed (Brimioulle et al. 2003), whereby maximum theoretical RV pressure (*P*_max_) is calculated from a non-linear extrapolation of the early systolic and diastolic portions of the RV pressure curve. End systolic pressure (ESP) was approximated by mean pulmonary artery pressure (mPAP) (Chemla et al. 1996). Ees was then calculated as the slope of the end-systolic pressure–volume (PV) relationship: (*P*_max_ − mPAP) divided by mean stroke volume for the 7 s capture (SV) (Trip et al. 2013). Ea was estimated by the mPAP/SV ratio (Brimioulle et al. 2003; Spruijt et al. 2015). RV contractile reserve (ΔEes) was defined as follows: Ees_Peak_/Ees_Rest_ × 100 as a percentage of baseline. The study was approved by the Partners Human Research Committee (IRB #2011P000272) and written consent was waved for this retrospective analysis.

### Statistical analysis

To estimate required sample sizes, a previous study examining RV exercise responses in two groups of patients with pulmonary hypertension was used which examined patients exercising at submaximal work levels (Hsu et al. 2016). This resulted in an estimated group size of 10 patients with 90% power to detect a difference in RV end-systolic elastance (Ees) at the 5% significance level. As our study used symptom-limited maximal exercise testing, we judged 8–10 patients as an appropriate group size to detect significant differences in RV elastance. Continuous variables were expressed as mean ± standard deviation and categorical variables were expressed as number of subjects and proportions, *n* (%). Haemodynamic data fitting a normal distribution was analysed by one-way ANOVA with Bonferroni correction to examine differences in means between three groups. Where data were non-normally distributed, comparisons of continuous variables were performed using the Wilcoxon–Mann–Whitney test, while Fisher’s exact test was used to compare categorical variables. Pearson correlation coefficients were used to compare relationships between RV coupling parameters and exercise haemodynamic variables and regression analysis was used to assess significance of any correlations. In all tests, a two-sided *p* value < 0.05 was considered significant. Statistical analysis and graphic creation were performed using Stata software Version 12 (Stata Corp LP, College Station, TX, USA).

## Results

Demographics, comorbidities and resting spirometry are displayed for control and obese groups in Table 1. Two out of eight patients in the Obese+ePVH group were in resting respiratory failure defined by a PaO_2_ < 60 mmHg and/or a PaCO_2_ > 45 mmHg. Both obese groups had a greater proportion of coronary risk factors and received greater amounts of vasoactive medications compared to controls. The Obese+ePVH group were older, had lower FEV_1_ values and received more antihypertensive treatments including beta blockade.

**Table 1:** Patients’ demographics and cardiovascular risk profiles

Patients’ characteristics	Controls (*n* = 6)	Obese−ePVH (*n* = 8)	Obese+ePVH (*n* = 8)
Age (years)	59 ± 5	51 ± 9	62 ± 9^c^
Female sex, *N* (%)	2 (33)	3(38)	3 (38)
Body mass index (kg/m^2^)	25.8 ± 2.3	37.0 ± 4.3^a^	38.4 ± 10.4^b^
Hypertension (%)	1	2	6^b,c^
Diabetes mellitus (%)	0	1	1
Coronary artery disease (%)	0	1	2^a^
Calcium channel blocker (%)	0	1	1
Beta blocker (%)	0	1	4^b,c^
ACE inhibitor or ARB (%)^d^	0	2^a^	2^b^
Diuretic (%)^e^	1	1	4^b,c^
FEV_1_%	100 ± 20	86 ± 22^a^	66 ± 9^b,c^
FEV_1_/FVC	78 ± 10	81 ± 4	77 ± 5
Hb (g/dL)	15.4 ± 0.8	13.6 ± 2.5	13.8 ± 2.7

^a^*p* < 0.05 controls vs Obese−eHFpEF

^b^*p* < 0.05 controls vs Obese+eHFpEF

^c^*p* < 0.05 Obese−eHFpEF vs Obese+eHFpEF

### Cardiopulmonary exercise data

Cardiopulmonary exercise data are presented in Table 2 showing lower absolute peak *V*O_2_ and peak workload in both Obese−ePVH and Obese+ePVH groups. Reduced peak *V*O_2_ in obesity was driven by both reduced O_2_ delivery and reduced systemic O_2_ extraction. Lower minute ventilation in the Obese+ePVH group resulted in increased end-tidal pCO_2_ at peak exercise. Three out of eight patients in the Obese−ePVH group and four out of eight (50%) patients in the Obese+ePVH group demonstrated a pulmonary mechanical limitation to exercise defined by peak minute ventilation/maximum voluntary ventilation > 70%. 0/6 (0%) in controls demonstrated pulmonary mechanical limitation by similar criteria. Maximal exercise testing was defined by attainment of either a RER > 1.05 or HR > 90% (predicted value) in all patients included in the final analysis.

**Table 2:** Non-invasive cardiopulmonary exercise data at rest and peak exercise

	Controls (*n* = 6)		Obese−ePVH (*n* = 8)		Obese+ePVH (*n* = 8)	
	Rest	Peak	Rest	Peak	Rest	Peak
*V*O_2_ (mL/kg/min)	–	30.2 ± 3.4	–	18.5 ± 7.7^d^	–	14.1 ± 3.5^e^
*V*O_2_ %	–	115 ± 14	–	88 ± 22	–	75 ± 21^e^
Work (W)	–	202 ± 36	–	148 ± 52^d^	–	81 ± 33^e,f^
RER	0.82 ± 0.11	1.16 ± 0.10	0.82 ± 0.08	1.15 ± 0.05	0.90 ± 0.13	1.09 ± 0.08
Lactate	0.7 ± 0.1	6.4 ± 1.5	1.1 ± 0.5	5.9 ± 2.8	1.6 ± 0.6^b^	4.7 ± 1.7^e^
HR (bpm)	68 ± 10	159 ± 5	74 ± 19	156 ± 18	80 ± 15	124 ± 22^e,f^
HR (%)	–	99 ± 5		92 ± 9	–	78 ± 13^e,f^
VE (L/min)	8 ± 2	66 ± 17	7 ± 2	63 ± 25	7 ± 2	42 ± 11^e,f^
VE/MVV%	–	56 ± 14		66 ± 19	–	75 ± 17
SaO_2_ (%)	98 ± 1	95 ± 2	95 ± 6	94 ± 3	95 ± 4	90 ± 9
PetCO_2_ (mmHg)	37 ± 5	44 ± 6	42 ± 8	47 ± 10	39 ± 9	46 ± 10
VE/VCO_2_ slope	–	25 ± 4		26 ± 6	–	26 ± 4

*RER* respiratory exchange ratio, *HR* heart rate, *VE* minute ventilation, *MVV* maximum voluntary ventilation, *SaO*_*2*_ arterial O_2_ saturation, *PetCO*_*2*_ end tidal CO_2_ tension, *Vd/Vt* physiological dead space fraction

^a^*p* < 0.05 controls (rest) vs Obese−ePVH (rest)

^b^*p* < 0.05 controls (rest) vs Obese+ePVH (rest)

^c^*p* < 0.05 Obese−ePVH (rest) vs Obese+ePVH (rest)

^d^Controls (peak) vs Obese−ePVH (peak)

^e^Controls (peak) vs Obese+ePVH (peak)

^f^Obese−ePVH (peak) vs Obese+ePVH (peak)

### Exercise haemodynamics and gas exchange data

Invasive pulmonary haemodynamic data at rest and peak exercise are summarised in Table 3. No patients met criteria for resting PAH defined by mean pulmonary artery pressure ≥ 25 mmHg and PVR > 240 dyn/s/cm^5^ at rest (Galie et al. 2016). The Obese+ePVH group exhibited the largest exercise rise in right atrial pressure, mean pulmonary artery pressure and pulmonary arterial wedge pressure with elevated peak PVR by recently established criteria (Oliveira et al. 2016). Compared to controls, both Obese−ePVH and Obese+ePVH groups developed raised PaCO_2_ at peak exercise although exercise hypoxaemia was mild and not significantly different to controls.

**Table 3:** Invasive cardiopulmonary exercise data at rest (upright) and peak exercise

	Controls (*n* = 6)		Obese−ePVH (*n* = 8)		Obese+ePVH (*n* = 8)	
	Rest	Peak	Rest	Peak	Rest	Peak
SBP (mmHg)	139 ± 9	210 ± 24	147 ± 18	197 ± 34	149 ± 13	196 ± 23
DBP (mmHg)	76 ± 7	89 ± 7	84 ± 11	90 ± 16	79 ± 10	88 ± 9
CO%	–	114 ± 22	–	113 ± 27	–	96 ± 29
DO_2_ (mL/min)	1078 ± 145	3551 ± 584	1134 ± 366	2994 ± 1045	1027 ± 306	2242 ± 364^e^
C(a-v)O_2_ (mL/dL)	6.2 ± 0.7	14.3 ± 1.7	6.2 ± 0.8	11.5 ± 2.5^d^	5.8 ± 1.0	11.1 ± 1.7^e^
Arterial pH	7.44 ± 0.02	7.34 ± 0.01	7.42 ± 0.04	7.32 ± 0.04	7.40 ± 0.04^a^	7.34 ± 0.02
Mixed venous pH	7.42 ± 0.02	7.21 ± 0.03	7.41 ± 0.04	7.24 ± 0.05	7.40 ± 0.03	7.26 ± 0.03^e^
PaO_2_ (mmHg)	97 ± 7	85 ± 14	91 ± 19	79 ± 15	84 ± 16	78 ± 9
PaCO_2_ (mmHg)	35 ± 4	37 ± 2	41 ± 8	42 ± 6^d^	42 ± 5^b^	44 ± 6^e^
PvO_2_ (mmHg)	36 ± 3	24 ± 2	34 ± 2	26 ± 2	35 ± 2	25 ± 3
PvCO_2_ (mmHg)	41 ± 4	69 ± 5	41 ± 8	69 ± 9	50 ± 8^b^	66 ± 6
P(A-a)O_2_ (mmHg)	8 ± 17	32 ± 13	15 ± 10	34 ± 12	14 ± 9	34 ± 18
RAP (mmHg)	1 ± 1	6 ± 2	6 ± 4^a^	10 ± 3^d^	6 ± 2^bc^	16 ± 5^e,f^
mPAP (mmHg)	13 ± 1	28 ± 6	18 ± 5^a^	36 ± 8^d^	20 ± 8^b,c^	47 ± 12^e,f^
PAWP (mmHg)	7 ± 3	13 ± 2	11 ± 2^a^	15 ± 4	11 ± 2^b,c^	26 ± 5^e,f^
SvO_2_ (%)	66 ± 2	29 ± 5	62 ± 6	32 ± 7	64 ± 4	33 ± 4
CO (L/min)	5.6 ± 1.1	17.0 ± 1.8	6.6 ± 1.5	17.1 ± 4.2	5.9 ± 2.3^c^	13.2 ± 2.7^e,f^
PVR (dyne/s/cm^5^)	108 ± 32	102 ± 18	119 ± 44	100 ± 35	133 ± 98	128 ± 61
PVC (mL/mmHg)	6.9 ± 2.3	3.7 ± 0.9	7.7 ± 2.8	3.2 ± 0.8	5.9 ± 2.6	2.4 ± 0.5^e,f^

*SBP* systolic blood pressure, *DBP* diastolic blood pressure, *CO* cardiac output, *DO*_*2*_ oxygen delivery, *C(a-v)O*_*2*_ arterio-venous oxygen difference, *PaO*_*2*_ arterial O_2_ tension, *PaCO*_*2*_ arterial CO_2_ tension, *PvO*_*2*_ mixed venous O_2_ tension, *PvCO*_*2*_ mixed venous CO_2_ tension, *P(A-a)O*_*2*_ Aa O_2_ gradient, *RAP* right atrial pressure, *mPAP* mean pulmonary artery pressure, *PAWP* pulmonary arterial wedge pressure, *SvO*_*2*_ mixed venous O_2_ saturation, *PVR* pulmonary vascular resistance, *PVC* pulmonary vascular compliance

^a^*p* < 0.05 controls (rest) vs Obese−ePVH (rest)

^b^*p* < 0.05 controls (rest) vs Obese+ePVH (rest)

^c^*p* < 0.05 Obese−ePVH (rest) vs Obese+ePVH (rest)

^d^Controls (peak) vs Obese−ePVH (peak)

^e^Controls (peak) vs Obese+ePVH (peak)

^f^Obese−ePVH (peak) vs Obese+ePVH (peak)

### RV–arterial coupling responses

**Fig. 2:**
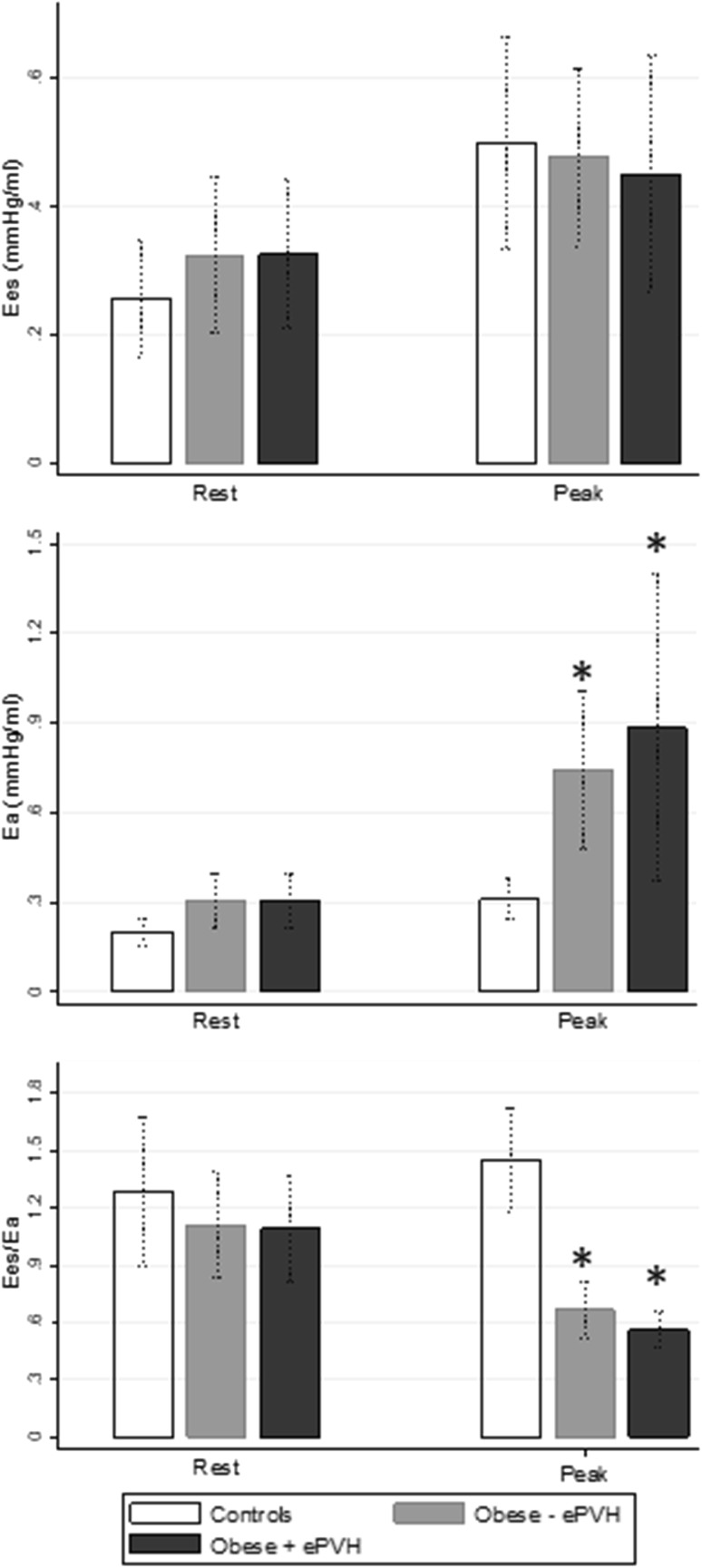
Comparison of RV arterial coupling parameters (Ees, Ea, Ees/Ea) at rest and peak exercise in controls (*n* = 6), Obese−ePVH (*n* = 8) and Obese+ePVH (*n* = 8) groups. Asterisk represents *p* < 0.05 for each obese group compared to controls at peak exercise

Rest and peak exercise RV contractility (Ees), arterial elastance (Ea) and RV–arterial coupling (Ees/Ea) data are displayed in Fig. 2 for controls, Obese−ePVH and Obese+ePVH groups. Ees and Ea at rest did not significantly differ between any group (Resting Ees = 0.26 ± 0.09 vs 0.32 ± 0.15 vs 0.33 ± 0.14 mmHg/mL, *p* = 0.56 (one-way ANOVA); resting Ea = 0.20 ± 0.04 vs 0.30 ± 0.11 vs 0.30 ± 0.11 mmHg/mL, *p* = 0.13). Resting Ees/Ea was also preserved across groups (Ees/Ea = 1.29 ± 0.37 vs 1.11 ± 0.33 vs 1.09 ± 0.33, *p* = 0.54, respectively).

**Fig. 3:**
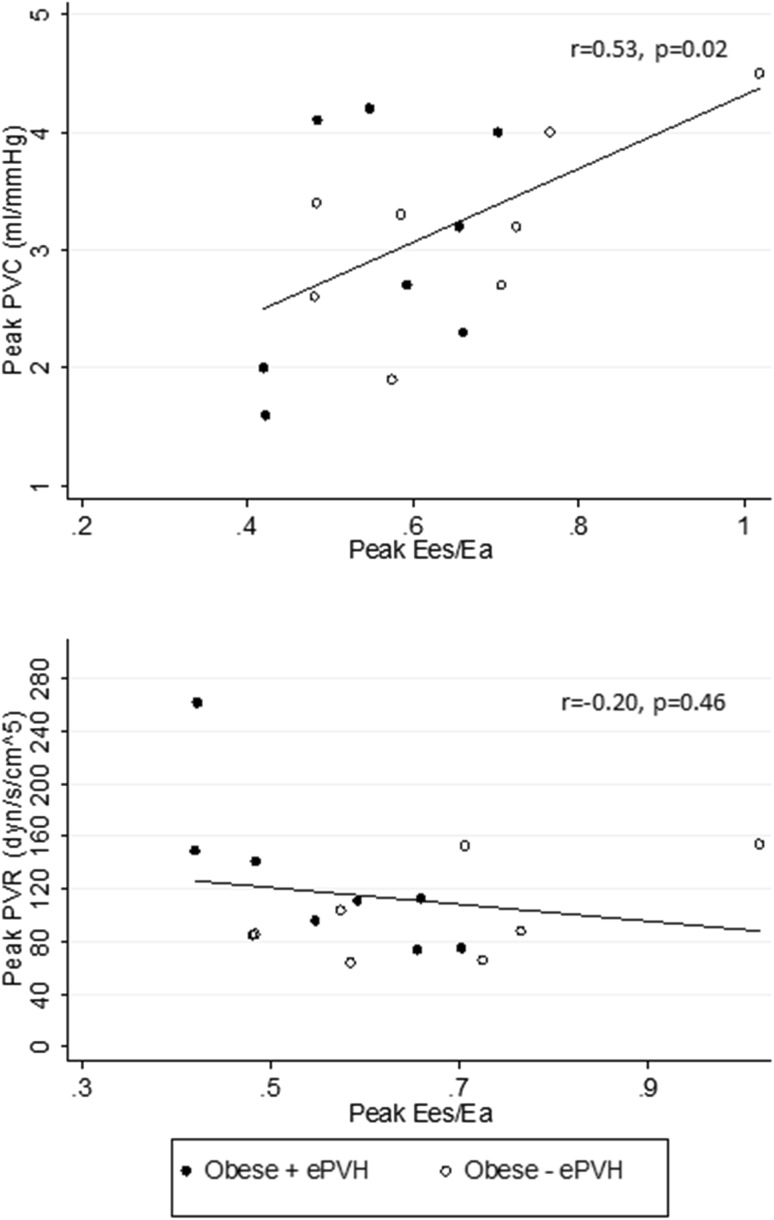
Relationships between peak Ees/Ea and both peak PVC (top) and peak PVR (bottom) in Obese−ePVH (*n* = 8) and Obese+ePVH (*n* = 8) groups

Between rest and peak exercise, Ea increased significantly across all groups (all *p* < 0.05). Peak Ees/Ea was preserved in controls and reduced in Obese−ePVH and Obese+ePVH groups to < 1.0 (peak Ees/Ea = 1.45 ± 0.26 vs 0.67 ± 0.18 vs 0.56 ± 0.11 respectively, *p* < 0.001). Reduced Ees/Ea was attributable to predominant increase in peak exercise Ea in obese groups (peak Ea = 0.31 ± 0.07 vs 0.75 ± 0.32 vs 0.88 ± 0.62 mL/mmHg respectively, *p* = 0.043) in the context of similar RV contractility (peak Ees = 0.50 ± 0.16 vs 0.45 ± 0.22 vs 0.48 ± 0.17 mL/mmHg, *p* = 0.89). Reduced exercise Ees/Ea did not differ between Obese−ePVH and Obese+ePVH groups (*p* = 0.65). Contractile reserve (ΔEes) was higher in controls than in Obese−ePVH and Obese+ePVH groups (ΔEes = 224 ± 80 vs 154 ± 39 vs 141 ± 34% of baseline, *p* < 0.001) with no difference between Obese−ePVH and Obese+ePVH groups (*p* = 0.78). Peak Ees/Ea positively correlated with peak PVC (*r* = 0.53, *p* = 0.02) but not with peak PVR (*r* = − 0.20, *p* = 0.46) (Fig. 3).

### Exercise haemodynamic responses in larger cohort

**Fig. 4:**
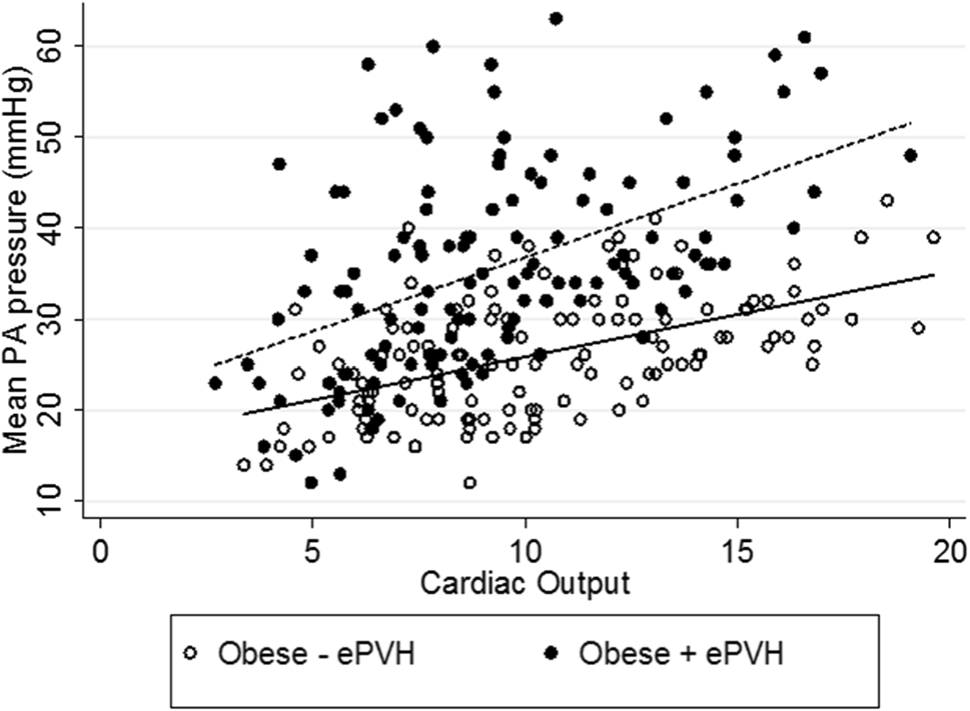
Minute by minute mPAP-cardiac output slopes in larger obese cohorts: Obese−ePVH (*n* = 20, 0.94 mmHg/L/min) and Obese+ePVH (*n* = 17, 1.63 mmHg/L/min) groups

**Fig. 5:**
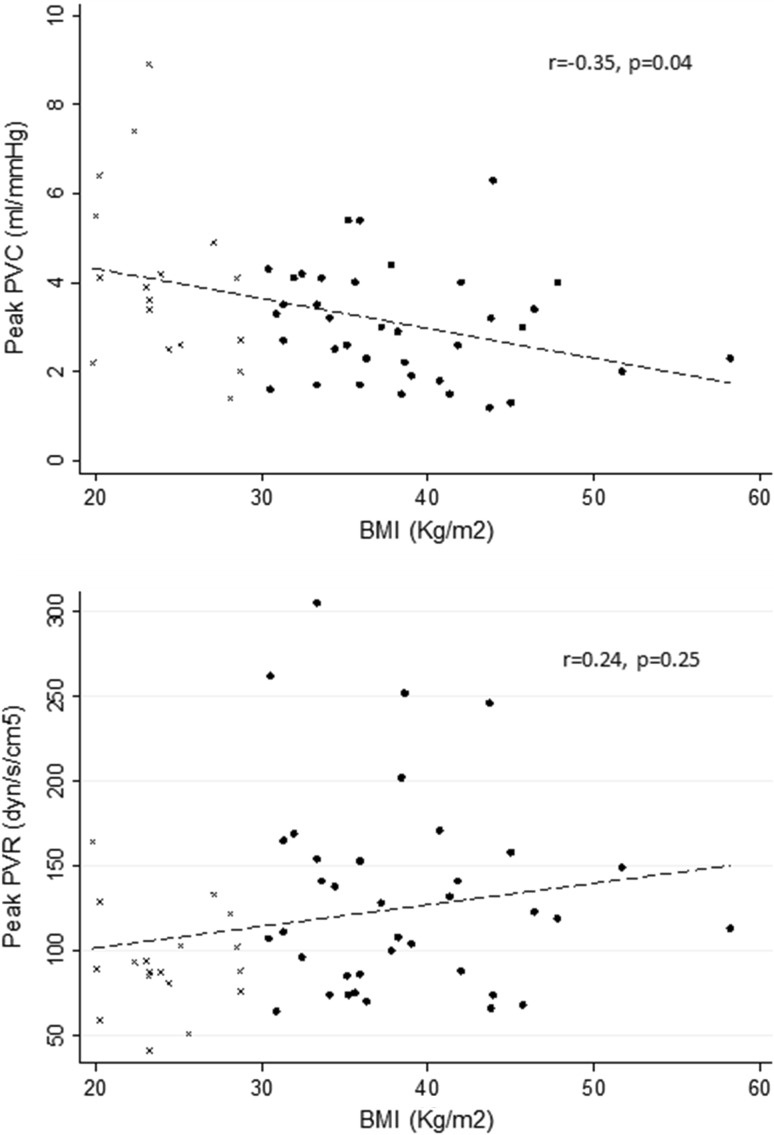
Relationship between BMI and peak exercise PVC (top) and peak exercise PVR (bottom) in controls (*n* = 18; small crosses) and obese patients (*n* = 37; black circles)

Table 4 compares exercise haemodynamic responses in the larger cohort of Control (*n* = 18) and obese patients (Obese−ePVH, *n* = 20; Obese+ePVH, *n* = 17). Compared to the Obese−ePVH group at peak exercise, the Obese+ePVH group developed higher right atrial pressure and lower cardiac output with reduced oxygen delivery. The lower cardiac output of the Obese+ePVH group was driven primarily by impaired exercise chronotropy given similar stroke volume augmentation. Mean pulmonary artery pressure-cardiac output responses in the larger cohort of both obese groups are shown in Fig. 4 and demonstrate a lower slope in the Obese−ePVH vs Obese+ePVH group (0.94 vs 1.63 mmHg/L/min, *p* < 0.05). The relationship between BMI and exercise PVC and exercise PVR are shown in Fig. 5. This shows a negative correlation between BMI and exercise PVC (*r* = − 0.35, *p* = 0.04) whereas there was no correlation between BMI and exercise PVR (*r* = 0.24, *p* = 0.25).

**Table 4:** Haemodynamic data from larger control and obese cohorts

	Controls (*n* = 18)		Obese−ePVH (*n* = 20)		Obese+ePVH (*n* = 17)	
	Rest	Peak	Rest	Peak	Rest	Peak
RAP (mmHg)	2 ± 2	4 ± 4	6 ± 3^a^	9 ± 3^d^	7 ± 3^b^	15 ± 4^e,f^
mPAP (mmHg)	11 ± 2	28 ± 7	18 ± 5^a^	35 ± 7^d^	21 ± 6^b^	46 ± 10^e,f^
PAWP (mmHg)	4 ± 2	11 ± 4	10 ± 2^a^	15 ± 3	11 ± 3^b^	26 ± 5^e,f^
CO (L/min)	4.8 ± 1.8	15.2 ± 4.2	5.9 ± 1.7^c^	14.6 ± 3.9	5.4 ± 2.0	11.3 ± 3.2^e,f^
PVR (dyne/s/cm^5^)	133 ± 41	94 ± 30	136 ± 60	116 ± 47	168 ± 100	150 ± 68^e^
PVC (mL/mmHg)	6.9 ± 3.6	4.1 ± 2.0	6.8 ± 2.5	3.3 ± 1.0	5.0 ± 2.1	2.6 ± 1.0^e,f^

^a^*p* < 0.05 controls (rest) vs Obese−ePVH (rest)

^b^*p* < 0.05 controls (rest) vs Obese+ePVH (rest)

^c^*p* < 0.05 Obese−ePVH (rest) vs Obese+ePVH (rest)

^d^Controls (peak) vs Obese−ePVH (peak)

^e^Controls (peak) vs Obese+ePVH (peak)

^f^Obese−ePVH (peak) vs Obese+ePVH (peak)

## Discussion

The principle observations from this study were of exercise uncoupling between the RV and pulmonary artery and a reduction in RV contractile reserve in symptomatic obese patients both of which occurred irrespective of changes in exercise LV filling pressure. RV–arterial uncoupling was driven primarily by impaired RV contractile responses to higher exercise RV afterload in obesity. This suggests that even in the absence of elevated LV filling pressures on exercise, the RV in obesity may undergo maladaptation at higher flow. Potential explanations for this include any combination of higher RV exercise afterload specific to obesity, metabolic dysregulation of pulmonary vascular tone or the presence of RV myocardial dysfunction intrinsic to obesity.

In the larger cohort, the obesity exercise response in patients demonstrating raised LV filling pressures on exercise was characterised by lower peak cardiac output, a steeper mean pulmonary artery pressure-flow response and lower pulmonary vascular compliance. This same group also harboured lower FEV1 values which may have also contributed to lower pulmonary vascular compliance and high exercise afterload. The positive relationship between higher BMI and lower pulmonary vascular compliance at peak exercise, when all pulmonary capacitance vessels are maximally recruited, further suggests that increasing severity of obesity may predispose to increased RV afterload on exercise.

Reduced RV–arterial coupling ratios in obesity signify a loss in energetic efficiency between forward blood flow from the RV to pulmonary artery. This lends further insight into the pathophysiology of exercise RV dysfunction in obesity in that elevated LV filling pressures, which did not influence RV exercise coupling, appear to play a less critical role in moderating RV exercise responses. This as well as the finding of lower RV contractile reserve supports the hypothesis of intrinsic RV dysfunction in obesity suggesting an increasing predisposition to obesity-associated pulmonary vascular dysfunction may occur at upper extremes of BMI.

One potential mechanism of RV contractile impairment in obesity may be higher circulating plasma volume which on exercise predisposes to exercise RV dilatation through higher venous return (Alpert et al. 2014; Obokata et al. 2017). Increased sympathetic nervous system activation and metabolic dysregulation may also drive higher filling pressures in obesity (Ketabchi et al. 2009; Noble et al. 1981). Both obese groups in our study developed higher right atrial pressure with exercise suggesting excess RV preload. In the Obese+ePVH group, right atrial pressure rose even further perhaps through higher LV filling pressures and greater atrial septal interaction. In support of this finding, Obokata et al. recently showed higher ventricular filling pressures led to greater ventricular interdependence in obesity through greater pericardial restraint (Obokata et al. 2017). Although we could not directly measure RV volume in our study, higher RV filling pressures are likely to drive greater exercise RV dilatation in turn increasing RV wall stress and mechanical inefficiency (Alpert et al. 1989). Thus, greater metabolic fatigue within a RV operating at higher ventricular volumes may account for reduced contractile responsiveness such as we observed in obesity.

Exercise pulmonary vascular responses in obesity demonstrated significant elevation in RV exercise afterload. Peak exercise LV filling pressures were highest in the Obese+ePVH group (by study design); however, the Obese−ePVH group who also demonstrated high exercise afterload (high Ea), had similar exercise pulmonary arterial wedge pressure values to controls. This suggests upstream transmission of high LV filling pressure on exercise insufficiently accounts for the higher afterload observed in this group. One plausible explanation for higher exercise afterload in obesity may be inadequate pulmonary vasodilatation. This has a number of potential origins including enhanced sympathetic signalling associated with obesity, increased predisposition to exercise-associated pulmonary vasoconstriction via the metaboreflex and older age (Lykidis et al. 2008). Alongside this, obesity phenotypes associated with the metabolic syndrome may also predispose to impaired pulmonary endothelial function via reduced nitric oxide availability driven by vasoactive adipokines (Lai et al. 2016; Yudkin et al. 2005). It seems likely therefore that either age-related, mechanical or metabolic factors exert the predominant influence over exercise RV afterload in obesity with a lesser contribution from increased LV filling pressures.

Obese patients with and without high exercise LV filling pressures differed in several important characteristics. The Obese+ePVH group were older, had greater exposure to vasoactive medications particularly beta blockers and had lower values for FEV_1_. In keeping with more severe exercise haemodynamic derangement and the older age of this group, LV compliance may have been reduced by increased prevalence of systemic hypertension and a longer duration of obesity (Alpert et al. 2014). Beta blockers may also have conceivably reduced RV contractile responses in four out of eight patients. Against this conclusion however was the finding of similar RV contractile reserve (ΔEes) in the Obese−ePVH group, where only one of eight patients took beta blockers. This makes a significant drug contribution of beta blockade in the Obese+ePVH group unlikely. Recent evidence suggests that reduction in tachycardia, observed in the Obese+ePVH group on exercise, may act to reduce RV pulsatile loading and thus total afterload (Metkus et al. 2016). We cannot therefore discount the possibility of both detrimental and beneficial effects of beta blockade on RV and pulmonary vascular exercise responses in our Obese+ePVH cohort.

Lower FEV_1_ values in the Obese+ePVH group gave rise to marked differences in ventilatory response with a greater proportion of patients in this group developing a pulmonary mechanical limitation to exercise. In contrast to typical gas exchange responses in HFpEF without obesity, the Obese+ePVH group developed lower O_2_ saturations and raised PaCO_2_ levels at peak exercise compared to controls, driven by inadequate compensatory hyperventilation. The net result is a lowering of the VE/VCO_2_ slope in obesity allowing for preserved VCO_2_ at a lower level of alveolar ventilation. We did not undertake an isoWork analysis to enable meaningful gas exchange comparisons between groups; however, higher arterial and, by implication, *alveolar* pCO_2_ levels have been shown to provoke greater pulmonary vasoconstriction (Barer and Shaw 1971; Kregenow and Swenson 2002; Nishio et al. 2001; Noble et al. 1981; Sweeney et al. 1998; Viitanen et al. 1990) which may have added to RV afterload in obesity. Dempsey and Wagner have previously highlighted the influence of a raised PaCO_2_ on exercise-induced hypoxaemia during maximal exercise (Dempsey and Wagner 1999). We found that exercise hypoxaemia was mild in both obese groups in our study, hence it is unlikely that gas exchange observations carried significant influence on hypoxic pulmonary vasoconstriction. Instead, lower FEV_1_ in the Obese+ePVH group may reflect either an underlying restrictive lung deficit or presence of occult airflow obstruction not captured by exclusion of patients with a reduced FEV_1_/FVC ratio < 70%. Restrictive lung function and dynamic hyperinflation are both prevalent in obesity and can increase RV afterload through increased pulmonary vascular resistance (Pinsky 2016) and higher cardiac filling pressures. However given lack of available lung volume data, we used electronic averaging of haemodynamic pressures throughout the respiratory cycle to standardise against respiratory variation when analysing group differences in RV contractile response (Boerrigter et al. 2014).

Our study’s findings of exercise RV–arterial uncoupling and impaired contractile reserve in obesity complements recent reports of exercise RV dysfunction in HFpEF and obesity-associated HFpEF in which RV exercise performance may be compromised by both reduced RV contractile reserve and higher exercise afterload (Borlaug et al. 2016; Borlaug and Obokata 2017; Obokata et al. 2017). Compared to these populations, our obese groups maintained a high/normal pulmonary vascular resistance and lower pulmonary vascular compliance suggesting exercise elevation in mean pulmonary artery pressure was more likely an independent obesity effect than simply a passive response to high LV filling pressure on exercise (Oliveira et al. 2016). The development of HFpEF on exercise is still an emerging concept with no current agreed haemodynamic definition in place. This is despite evidence for both ‘passive’ and ‘reactive’ forms recently shown to carry prognostic relevance (Huang et al. 2017). Nevertheless, our data show exercise RV–arterial uncoupling in obese patients constitutes an abnormal pulmonary vascular response to exercise with the most significant contributor to RV afterload being a reduction in exercise pulmonary vascular compliance.

### Limitations

Our study was retrospectively conducted and so represented a highly selected group of symptomatic obese patients with relatively low levels of co-morbidity which may limit extrapolation to all obese patients, especially those with fewer symptoms. Specifically, the administration of medication to treat systemic hypertension may have modified RV contractile and afterload responses in an unpredictable manner. Our single-beat model also included presumptive use of mean pulmonary artery pressure as the end-systolic point of the RV pressure–volume relationship, which may lead to overestimation of contractility and afterload. As right atrial pressure also increases with exercise, the presumptive use of mPAP/mean SV likely underestimated the RV–arterial coupling ratios reported (Spruijt et al. 2015). Finally, the cross-sectional design of this study precludes insight into time-varying pathophysiology at different disease stages with the associated risk of Type 1 error from multiple statistical hypotheses.

## Conclusions

The contribution of central haemodynamics to exercise limitation and in particular, elevated left ventricular filling pressures have been studied with invasive haemodynamics for over 40 years in obesity (Alpert et al. 1989; Kaltman and Goldring 1976). High circulating blood volume is thought to underlie the high cardiac output state at rest in obesity, but exercise cardiac output has been shown to fall to low/normal levels especially at higher workloads in obesity. Exercise responses in obese patients demonstrated compromise in both central and peripheral components namely low oxygen delivery and low peripheral oxygen extraction. This contrasts with responses seen in HFpEF where oxygen extraction is typically increased in response to low cardiac output. In isolated HFpEF, exercise left ventricular filling has been shown to depend heavily on the myocardial reserve of both the left and right ventricle (Hussain et al. 2016), however greater biventricular interactions in obesity due to higher chamber volumes and greater pericardial restraint may have a deleterious effect. Our data support right ventricular contractile impairment arising due to higher pulmonary afterload although an intrinsic obesity effect is also implicated. Interplay between mediators of higher right ventricular afterload, increased intrathoracic pressure and elevated body mass index should therefore be further evaluated.

## Abbreviations

C(a-v)O_2_: Arteriovenous O_2_ content difference
CI: Cardiac index
CO: Cardiac output
DO_2_: Oxygen delivered
dPAP: Diastolic pulmonary artery pressure
Ea: Effective pulmonary arterial elastance
Ees: End systolic RV elastance
Ees/Ea: RV ventriculo–arterial coupling ratio
HFpEF: Heart failure with preserved ejection fraction
LV: Left ventricle
mPAP: Mean pulmonary artery pressure
PAWP: Pulmonary arterial wedge pressure
PVR: Pulmonary vascular resistance
PVC: Pulmonary vascular compliance
PaO_2_: Partial pressure of arterial O_2_
PAO_2_: Partial pressure of alveolar O_2_
PCO_2_: Partial pressure of carbon dioxide
PvO_2_: Partial pressure of mixed venous O_2_
PvCO_2_: Partial pressure of mixed venous CO_2_
PaCO_2_: Partial pressure of arterial CO_2_
RV: Right ventricle
VE: Minute ventilation
